# Supplementary material for: Role of IgG against N-protein of SARS-CoV2 in COVID19 clinical outcomes
Source: Sci Rep. 2021 Feb 10;11:3455. doi: 10.1038/s41598-021-83108-0 (PMC7875990; doi:10.1038/s41598-021-83108-0)
Supplement: Supplementary file 1 — Supplementary Information. [file 41598_2021_83108_MOESM1_ESM.docx]

**Role of IgG against N-protein of SARS-CoV2 in COVID19 clinical outcomes**

Mayank Batra1, Runxia Tian1, Chongxu Zhang1, Emile Clarence2, Camila Sofia Sacher2, Justin Nestor Miranda2, Justin Rafa O De La Fuente2, Megan Mathew2, Desmond Green2, Sayari Patel2, Maria Virginia Perez Bastidas1, Sara Haddadi1, Mukunthan Murthi1, Miguel Santiago Gonzalez1, Shweta Kambali1, Kayo H M Santos1, Huda Asif1,Farzaneh Modarresi3, Mohammad Faghihi3, and Mehdi Mirsaeidi1*

1Division of Pulmonary and Critical Care, University of Miami, Miami, FL

2School of Medicine, University of Miami, Miami, FL

3Express Gene Laboratory, Miami, FL

**Including:**

1. Table S1. Characteristics of 400 patients with RT-PCR confirmed COVID19 enrolled in the study
2. Table S2. Demographic characteristics of patients with COVID19 that were admitted to MICU
3. Table S3. Comorbidities and outcomes of patients with COVID19 that admitted in MICU
4. Table S4. Demographic characteristics of patients who died during hospitalization
5. Table S5. Laboratory characteristics of patients with COVID19 that died during hospitalization

**Table S1**. Characteristics of 400 patients with RT-PCR confirmed COVID19 enrolled in the study

| Variables | Hospitalized | | P value, OR, CI |
| --- | --- | --- | --- |
|  | Yes  Number (%) | No  Number (%) |  |
| Male | 141 (56.9) | 107 (43.1) | 0.004, 1.84 (1.22-2.77) |
| Age (years) M±SD | 63.16 ± 17.22 | 47.30 ± 14.57 | <0.0001 |
| Height (cm) M±SD | 166.47 ± 21.83 | 169.30 ± 10.36 | 0.117 |
| Weight (lb.) M±SD | 179.42 ± 45.19 | 188.46± 46.39 | 0.074 |
| European American | 147 (61) | 102 (64.2) | 0.524 |
| Hispanic | 153 (67.4) | 86 (58.1) | 0.068 |
| African American | 62 (70.5) | 187 (59.9) | 0.074 |
| CCI | 3.22(2.48) | 1.21(1.59) | <0.0001 |

*OR and CI were calculated for variables with P value <0.05.

**Table S2**. Demographic characteristics of patients with COVID19 that were admitted to MICU

| Variables | MICU admission | | P value, OR^*^, CI^*^ |
| --- | --- | --- | --- |
|  | **Yes**  **Number (%)** | **No**  **Number (%)** |  |
| Male | 50 (65.8) | 93 (52.5) | 0.053, 1.74 (0.99-3.04) |
| Age (years) M±SD | 65.70 ± 14.81 | 61 ± 18.08 | 0.079 |
| Height (cm) M±SD | 167.57 ± 10.60 | 165.91 ± 21 | 0.516 |
| Weight (lb.) M±SD | 185.59 ± 44.26 | 177.06 ± 45.39 | 0.170 |
| Patient delay (days) | 4.49 ± 4.41 | 4.30 ± 3.94 | 0.768 |
| Doctor delay (days) | 4.25 ± 4.36 | 4.14 ± 3.81 | 0.860 |
| Current smoker | 5 (6.6) | 13 (7.6) | 0.766 |
| Current alcohol ingestion | 22 (31.4) | 45 (26.9) | 0.485 |
| Current Marijuana ingestion/inhalational | 2(3.5) | 6 (4.1) | 0.843 |
| Nursing Home | 12 (16) | 29 (16) | 0.921 |
| Medication intake prior hospital admission |  |  |  |
| Chemotherapy | 10 (13.5) | 9 (5.3) | 0.033, 2.8 (1.085-7.2) |
| Inhaled corticosteroid | 10 (13.3) | 18 (10.8) | 0.566 |
| Ace inhibitors | 15 (20) | 27 (16) | 0.443 |
| Angiotensin Receptor Blockers | 9 (12) | 24 (14.2) | 0.643 |
| Statins | 27 (36) | 53 (31.4) | 0.477 |
| CT or Chest Xray findings obtained after hospital Admission |  |  |  |
| Ground glass opacities | 15 (19.7) | 45 (26.5) | 0.257 |
| Consolidation | 22 (28.9) | 25 (14.8) | 0.01, 2.35 (1.22-4.51) |
| Bilateral infiltrates | 45 (59.2) | 76 (44.7) | 0.036, 1.8 (1.04-3.11) |
| Laboratory Findings within 48 hours of hospital admission |  |  |  |
| Leukocytes (10^3^/uL) | 9.49 ± 6.99 | 7.66 ± 4.07 | 0.036 |
| Neutrophils (10^3^/uL) | 10.99 ± 14.82 | 8.73 ± 14.38 | 0.259 |
| Lymphocytes (10^3^/uL) | 2.11 ± 4.75 | 1.96 ± 3.56 | 0.779 |
| Eosinophils (10^3^/uL) | 0.19 ± 0.9 | 0.07 ± 0.18 | 0.311 |
| Hemoglobin (g/dL) | 13.63± 15.25 | 12.28 ±2.51 | 0.253 |
| Hematocrit (%) | 36.63± 7.52 | 37.43 ± 7.73 | 0.444 |
| Platelet count (10^3^/uL) | 199.86 ± 128.41) | 234 ± 105.94 | 0.025 |
| Interleukin-6 (pg/mL) | 572.09 ± 938.80 | 108.87 ± 228.24 | 0.011 |
| C-Reactive Protein (mg/dL) | 13.84 ± 10.43 | 9.84 ± 11.38 | 0.017 |
| Erythrocyte sedimentation rate (mm/hr) | 48.79 ± 29.42 | 43.42 ± 30.63 | 0.461 |
| Blood urea nitrogen (mg/dL) | 28.06 ± 23.40 | 21.74 ± 19.32 | 0.041 |
| Creatinine (mg/dL) | 2.11 ± 2.83 | 1.48 ± 1.80 | 0.079 |
| Ferritin (ng/mL) | 1436.29 ± 2403.75) | 1113.80 ± 1950.25 | 0.326 |
| Procalcitonin (ng/mL) | 5.21± 23.82 | 2.66 ± 13.81 | 0.380 |
| Lactate (mmol/L) | 11.44 ± 70.70 | 14.51 ± 81.43 | 0.792 |
| Troponin (ng/mL) | 42.34 ± 282.52 | 0.25 ± 0.45 | 0.322 |
| Creatine kinase (U/L) | 303.08 ± 421.19 | 254.86 ± 491.27 | 0.564 |
| Lactate dehydrogenase (U/L) | 507.47 ± 367.86 | 374.45± 195.87 | 0.01 |
| Fibrinogen (mg/dL) | 584.89 ± 132.19 | 528.33± 214.70 | 0.536 |
| ALT (U/L) | 85.39 ± 206.10 | 51.16 ± 73.40 | 0.178 |
| AST (U/L) | 143.69 ± 632.69 | 58.27 ± 65.6 | 0.260 |
| Albumin (mg/dL) | 3.39 ± 0.73 | 3.68 ± 0.50 | 0.003 |
| Bilirubin (mg/dL) | 0.59 ± 0.34 | 0.67 ± 1.21 | 0.830 |
| D-dimer (ug/mL) | 4.14 ± 5.55 | 5.29 ± 28.69 | 0.797 |

*OR and CI were calculated for categorical variables with P value <0.05, M±SD: Mean± standard deviation, ALT: Alanine aminotransferease, AST: Aspartate aminotransaminase

**Table S3**. Comorbidities and outcomes of patients with COVID19 that admitted in MICU

| Variables | MICU admission | | P value, OR*, CI* |
| --- | --- | --- | --- |
| Comorbidities | Yes Number (%) | No Number (%) |  |
| Asthma | 6 (8.2) | 24 (14) | 0.211 |
| Pulmonary embolism | 4 (5.4) | 4 (2.3) | 0.222 |
| COPD | 6 (8.1) | 15 (8.7) | 0.885 |
| Emphysema | 2 (2.7) | 3 (1.7) | 0.623 |
| Chronic heart failure | 6 (8.1) | 9 (5.3) | 0.397 |
| Coronary Artery Disease | 5 (6.8) | 21 (12.2) | 0.209 |
| Atrial fibrillation | 8 (10.8) | 16 (9.2) | 0.694 |
| Hypertension | 50 (67.6) | 106 (60.2) | 0.275 |
| Stroke | 6 (8.3) | 13 (7.4) | 0.808 |
| Dementia | 5 (6.8) | 21 (11.9) | 0.238 |
| Chronic Renal Failure | 11 (15.1) | 16 (9.1) | 0.176 |
| Liver Disease | 1 (1.4) | 7 (4.0) | 0.308 |
| Diabetes Mellitus | 35 (47.3) | 47 (27.0) | 0.002, 2.42 (1.37-4.27) |
| Clinical Symptoms |  |  |  |
| Cough | 46 (67.6) | 101 (57.4) | 0.143 |
| Sputum | 5 (7.9) | 18 (10.5) | 0.556 |
| Chest pain | 4 (6.3) | 30 (17.4) | 0.037, 0.31 (0.10-0.93) |
| Dyspnea | 53 (73.6) | 104 (59.12) | 0.033, 1.93 (1.05-3.53) |
| Hemoptysis | 1 (1.5) | 3 (1.7) | 0.906 |
| Fever | 53 (73.6) | 105 (59.7) | 0.028, 1.99 (1.07-3.67) |
| Chills | 24 (36.4) | 51 (29.7) | 0.319 |
| Headache | 4 (6.2) | 17 (9.9) | 0.372 |
| Myalgia | 17 (25.8) | 43 (24.9) | 0.886 |
| Abdominal pain | 4 (6.3) | 29 (16.9) | 0.04, 0.32 (0.11-0.97) |
| Diarrhea | 11 (16.9) | 33 (19.2) | 0.69 |
| Nausea or vomiting | 10 (15.4) | 28 (16.2) | 0.881 |
| Altered Mental Status | 11 (16.4) | 19 (10.7) | 0.231 |
| Outcomes |  |  |  |
| ICU Death | 18 (24.3) | 1 (0.6) | <0.001, 56.250 (7.34-430.88) |
| Hospital death | 22 (29.7) | 9 (5.1) | <0.001, 7.85 (3.40-18.10) |

*OR and CI were calculated for variables with P value <0.05.

**Table S4**. Demographic characteristics of patients who died during hospitalization

|  | Hospital death | | P value, OR*, CI8 |
| --- | --- | --- | --- |
|  | Yes | No |  |
| Male | 22 (71.0) | 118 (54.1) | 0.082 |
| Hispanic | 21 (75.0) | 135 (63.7) | 0.242 |
| European American | 21 (67.7) | 126 (57.5) | 0.283 |
| African American | 4 (12.9) | 57 (26.0) | 0.121 |
| Smoking | 1 (3.4) | 16 (7.4) | 0.44 |
| Alcohol | 4 (15.4) | 62 (30.0) | 0.13 |
| Nursing home | 12 (38.7) | 26 (11.9) | <0.001, 4.68 (2.04-10.75) |
| Chemotherapy | 3 (10.3) | 15 (7.1) | 0.538 |
| Inhaled steroid | 3 (10.3) | 23 (11.0) | 0.915 |
| Ace inhibitors | 9 (31.0) | 32 (15.2) | 0.038, 2.51 (1.05-6.02) |
| Angiotensin receptor blocker | 6 (20.7) | 27 (12.8) | 0.252 |
| Statins | 12 (41.4) | 65 (30.8) | 0.256 |
| Ground glass | 6 (19.4) | 54 (25.6) | 0.455 |
| Consolidation | 13 (41.9) | 34 (16.2) | 0.001, 3.73 (1.67-8.34) |
| Bilateral infiltrates | 17 (54.8) | 103 (48.8) | 0.532 |

*OR and CI were calculated for variables with P value <0.05.

**Table S5.** Laboratory characteristics of patients with COVID19 that died during hospitalization

| Variables | Died | Survived | P value |
| --- | --- | --- | --- |
|  | Mean ± SD | Mean ± SD |  |
| Age (years) | 78.19±10.68 | 60.58±16.79 | <0.0001 |
| Height (in cm) | 161.20±31.70 | 167.04±15.9 | 0.106 |
| Weight (in lb) | 173.93±25.78 | 180.37±47.41 | 0.263 |
| Patient delay (days) | 3.67±4.04 | 4.46±4.1 | 0.432 |
| Doctor delay (days) | 2.50±2.62 | 2.5±2.62 | 0.058 |
| Leukocytes (10^3^/uL) | 11.75±7.69 | 7.74±4.59 | 0.008 |
| Neutrophils (10^3^/uL) | 13.35±14.93 | 8.6±13.6 | 0.074 |
| Lymphocytes (10^3^/uL) | 2.33± 5.17 | 1.91±3.71 | 0.577 |
| Eosinophils (10^3^/uL) | 0.36±1.38 | 0.07±0.18 | 0.293 |
| Hemoglobin (g/dL) | 12.00±2.36 | 12.82±9.24 | 0.627 |
| Hematocrit (%) | 37.25±7.48 | 37.9±7.34 | 0.921 |
| Platelet count (10^3^/uL) | 210.00±138.88 | 227.4±110.23 | 0.428 |
| Interleukin-6 (pg/mL) | 1228.64±1250.46 | 124.2±222.27 | 0.011 |
| C-Reactive Protein (mg/dL) | 13.11±9.48 | 10.85±11.5 | 0.326 |
| Erythrocyte sedimentation rate (mm/hr) | 47.75±35.48 | 45.3±29.4 | 0.801 |
| Blood urea nitrogen (mg/dL) | 39.37±22.97 | 21.25±19.56 | 0.000 |
| Creatinine (mg/dL) | 2.23±2.09 | 1.59±2.20 | 0.129 |
| Ferritin (ng/mL) | 2292.22±3600.14 | 1060.5±1742.9 | 0.105 |
| Procalcitonin (ng/mL) | 3.34±7.01 | 3.44±19.46 | 0.980 |
| Lactate (mmol/L) | 48.84±172.95 | 5.42±34.24 | 0.181 |
| Troponin (ng/mL) | 105.81±448.27 | 0.22±0.036 | 0.331 |
| Creatine kinase (U/L) | 438.37±567.66 | 242.8±452.3 | 0.087 |
| Lactate dehydrogenase (U/L) | 606.00±468.68 | 393.27±224.11 | 0.043 |
| Fibrinogen (mg/dL) | 656.00±153.10 | 538.8±165.7 | 0.288 |
| ALT (U/L) | 107.38±290.70 | 55.4±85.8 | 0.347 |
| AST (U/L) | 258.66±983.07 | 59.26±70.67 | 0.284 |
| Albumin (mg/dL) | 3.19±0.78 | 3.64±0.55 | 0.006 |
| Bilirubin (mg/dL) | 0.56±0.33 | 0.66±1.1 | 0.625 |
| D-dimer (ug/mL) | 5.35±6.25 | 4.85±25.40 | 0.936 |

*OR and CI were calculated for categorical variables with P value <0.05, M±SD: Mean± standard deviation, ALT: Alanine aminotransferease, AST: Aspartate aminotransaminase
